# Case Report: Steroid-responsive recurrent iron-deficiency anemia in pediatric collagenous gastritis

**DOI:** 10.3389/fped.2026.1969954

**Published:** 2026-09-23

**Authors:** Jia-ke Yu, Wen-jing Wang, Yi Chen, Hai Cao, Fang Liu, Huan-ying Yao, Wei-rong Liu

**Affiliations:** 1 Department of Pediatrics, Shaoxing People’s Hospital, The First Affiliated Hospital, Shaoxing University, Shaoxing, China; 2 Department of Pathology, Shaoxing People’s Hospital, The First Affiliated Hospital, Shaoxing University, Shaoxing, China

**Keywords:** collagenous gastritis, corticosteroids, hematologic remission, iron-deficiency anemia, pediatric

## Abstract

Collagenous gastritis (CG) is a rare inflammatory disorder of the stomach and an uncommon but important cause of iron-deficiency anemia in children. Currently, there are no accepted therapeutic guidelines for treating these patients. We report a case in an 11-year-old boy who presented with recurrent iron-deficiency anemia requiring blood transfusion with an ultimate diagnosis of CG on repeat endoscopy. While iron supplementation and proton pump inhibitors failed to achieve clinical remission, the addition of oral prednisone effectively corrected the anemia. Notably, endoscopy improved despite persistent histologic damage. Our case demonstrates that CG should be considered in children with recurrent iron-deficiency anemia. It also underscores the potential role of corticosteroids in the treatment of refractory patients, which can achieve clinical remission even without histological improvement.

## Introduction

Collagenous gastritis (CG) is a rare but significant diagnosis that has been increasingly recognized as an important, yet frequently overlooked, cause of iron-deficiency anemia (IDA) in children (1). Histologically, CG is characterized by the deposition of thickened subepithelial collagen, typically defined as collagen bands greater than 10 μm. This condition is also associated with inflammatory infiltrates in the gastric lamina propria (2). Pediatric CG typically presents with isolated gastric involvement and symptoms like abdominal pain, vomiting, fatigue, pallor, and IDA, making it challenging to diagnose patients with CG (3). Current treatment usually involves iron supplementation, proton pump inhibitors (PPI), corticosteroids, dietary restrictions on allergenic foods, and careful follow-up. However, no accepted therapeutic standards have been established, and different treatment strategies exhibit variable outcomes (4).

We report a case of pediatric CG that was refractory to initial therapy with iron and PPI, which resulted in recurrent blood transfusion and hospitalizations, and ultimately required step-up therapy for hematologic remission and endoscopic improvement.

## Case report

An 11-year-old boy with 1 week of worsening anemia and fatigue presented to the hospital after a vomiting event. Initial complete blood count showed hemoglobin 6.9 g/dL, hematocrit 23.6%, mean corpuscular volume 62.9 fL, and red cell distribution width 18.5%. The patient was subsequently admitted for a blood transfusion and to determine the cause of the anemia. He received 2 units of packed red blood cells (pRBC) in his first 24 h, which improved his fatigue. He previously demonstrated good health, age-appropriate growth and weight (height: 157 cm; weight: 50 kg; BMI: 20.2 kg/m^2^), and no other nutrient deficiencies and no history of food allergies. Hematology workup showed normal levels of lactate dehydrogenase, creatinine, urea, erythrocyte sedimentation rate, and C-reactive protein. The results of the thalassemia gene mutation test were negative. Serum iron levels of 2.2 μmol/L, total iron-binding capacity of 75.6 μmol/L, transferrin saturation of 3.3%, and ferritin of 5.3 ng/mL all indicated IDA, according to iron tests. The patient was then referred to the pediatric gastroenterologist for further workup. The differential for his presentation included celiac disease, eosinophilic gastritis, autoimmune gastritis, inflammatory bowel disease, and *Helicobacter pylori* gastritis. The coeliac serology and stool evaluation for fecal hemoccult blood and calprotectin were all negative. The patient underwent both esophagogastroduodenoscopy (EGD) and colonoscopy. EGD showed erosive nodular gastritis in the antrum and body, with biopsies taken from the antrum only. Histology confirmed chronic, mild, active inflammation, and the *Helicobacter pylori* test was negative. Colonoscopy revealed no significant findings. The patient was discharged with oral iron 150 mg once daily, omeprazole 20 mg once daily, and outpatient follow-up.

Two months later, the patient was re-admitted due to a hemoglobin level of 6.8 g/dL and worsening symptoms of dizziness and fatigue, despite reported adherence to oral iron supplementation. He was given two units of pRBC and intravenous sucrose infusions. The patient and his parents have no history of autoimmune diseases. He had not previously undergone autoantibody testing for autoimmune gastritis, both anti-parietal cell antibodies (APCA) and intrinsic factor antibodies were within normal limits. Repeat EGD revealed diffuse nodularity, thickened mucosal folds, and scattered erosions in the gastric body (Figure 1A), and the duodenal bulb and descending were normal. Histopathology revealed gastric body (Figures 1B,C) with active chronic gastritis, thickened subepithelial collagen bands, and 3 eosinophils/hpf in the lamina propria (LP). Duodenal biopsies revealed no signs of duodenitis. The patient's clinical history and histological findings support a diagnosis of CG. His laboratory values improved, with his hemoglobin increasing to 11.7 g/dL, ferritin increasing to 12.9 ng/mL, and iron levels increasing to 7.5 μmol/L. The patient was continued on supplementary oral iron and omeprazole, with outpatient follow-up.

**Figure 1:**
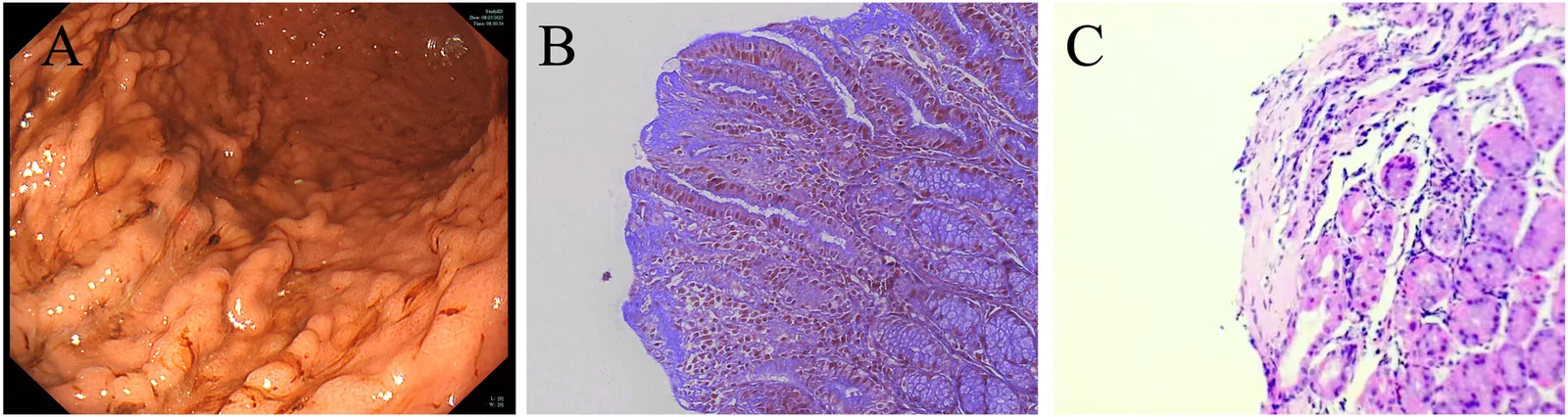
**(A)** Endoscopic images of the gastric body: shows diffuse nodularity, thickened mucosal folds, and scattered erosions. **(B)** Histopathological examination in gastric body: positive Masson trichrome stain (200× magnification) for subepithelial collagen deposition. **(C)** Histopathological examination in gastric body: hematoxylin and eosin stain (200× magnification) for 3 eosinophils/hpf in the lamina propria.

On follow-up 3 months later, the patient complained of mild abdominal pain, and his hemoglobin level dropped to 10.2 g/dL again. Repeat EGD demonstrated persistent erythematous nodular gastropathy, along with histologically persistent subepithelial collagen in biopsied sections. Given his current condition, he was subsequently placed on prednisone at a dosage of 15 mg twice daily (0.6 mg per kilogram), in addition to his omeprazole and supplemental iron. 8 weeks later, following clinical improvement and the patient's request, prednisone was weaned to 10 mg for 8 weeks. After 8 weeks of 10 mg prednisone, labs showed Hb at 13.9 g/dL. Follow-up endoscopies revealed endoscopic improvement in the gastric body, with improved nodularity and erythema (Figure 2A). Histopathology was virtually unchanged in the gastric body (Figure 2B), with a positive Masson trichrome stain for subepithelial collagen deposition. The patient ultimately stopped the prednisone and was able to reduce his iron supplementation and omeprazole gradually. He remains in good health. A timeline summarizing the diagnostic and therapeutic interventions is provided in Figure 3.

**Figure 2:**
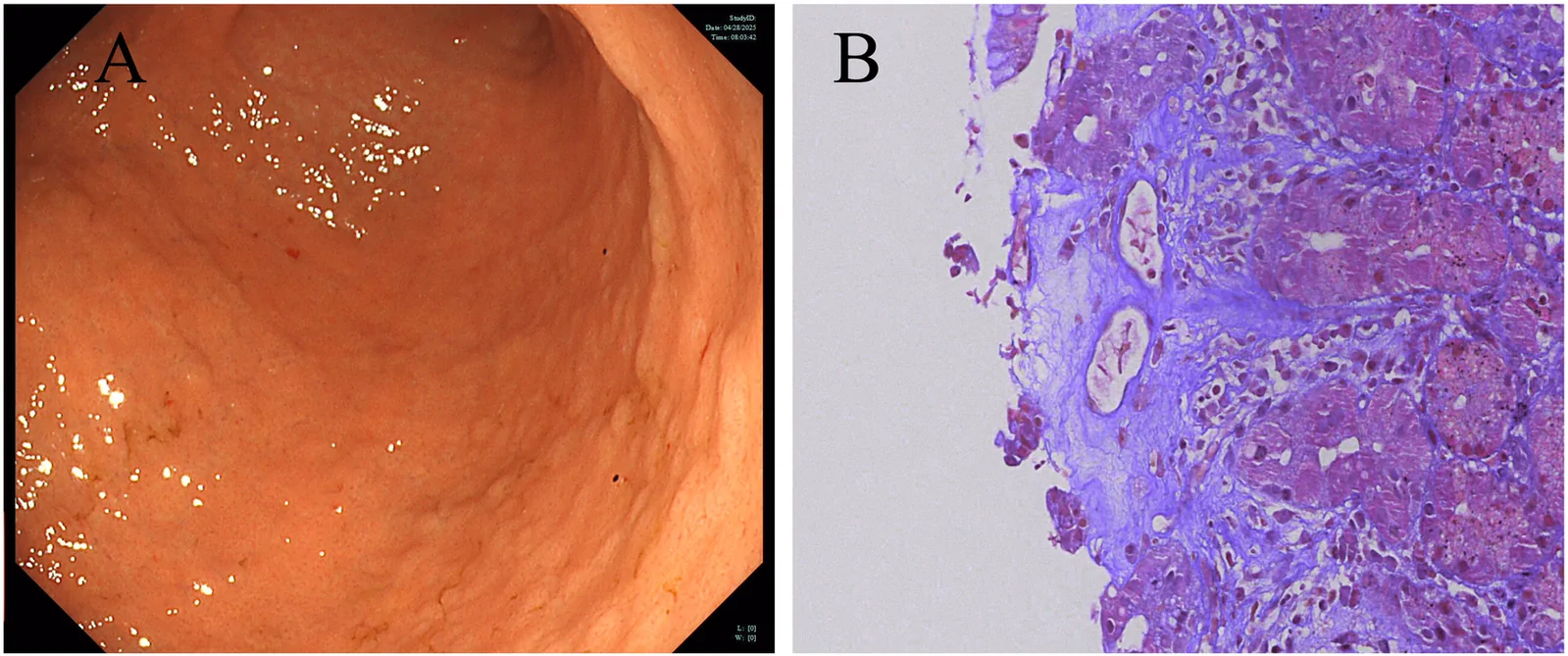
**(A)** Follow-up endoscopy after 16 weeks of prednisone revealed endoscopic improvement in the gastric body, with improved nodularity and erythema, **(B)** histopathological examination after 16 weeks of prednisone showed virtually unchanged in the gastric body, with a positive Masson trichrome stain for subepithelial collagen deposition.

**Figure 3:**
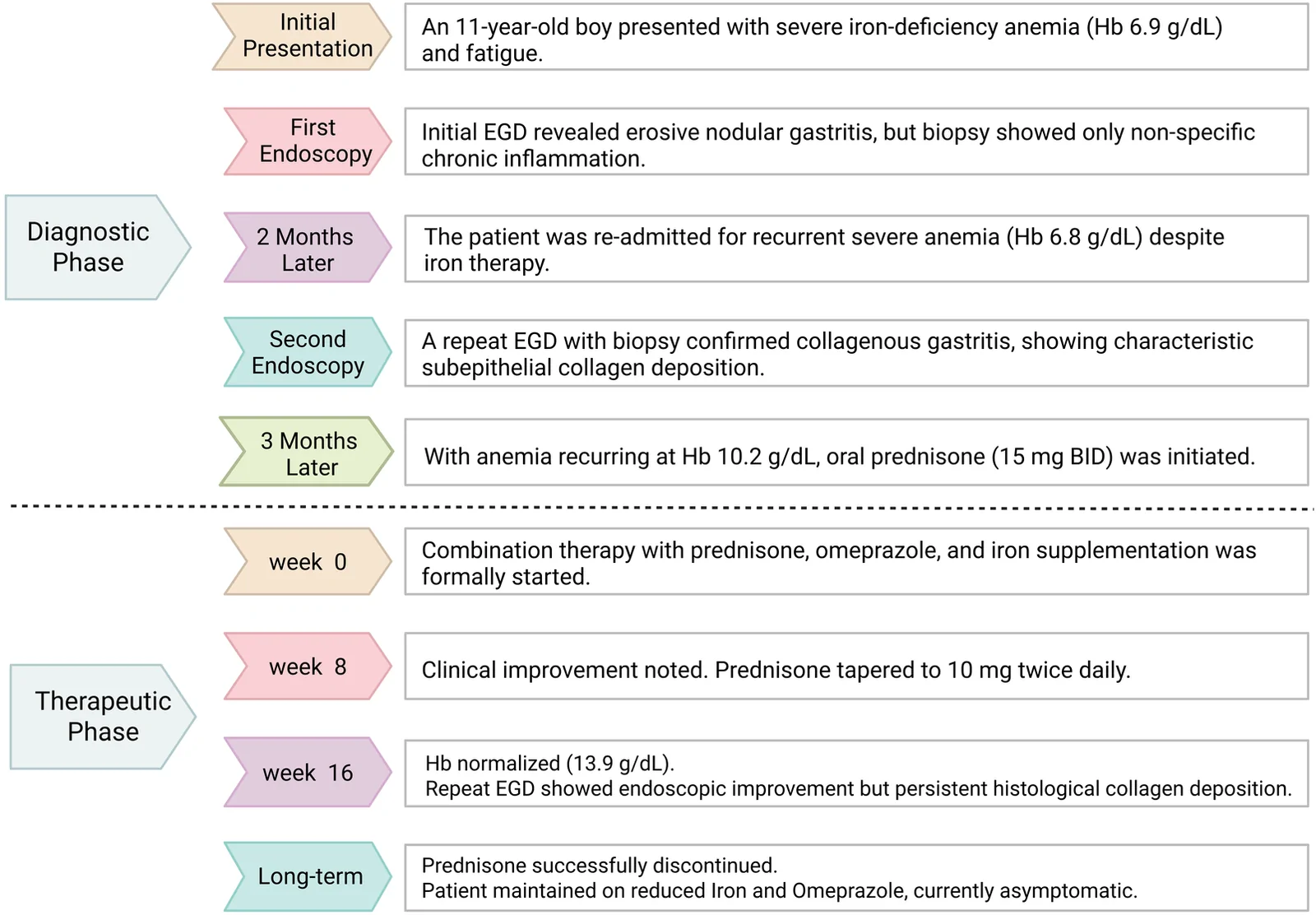
Timeline of the diagnostic and therapeutic interventions. It illustrates the clinical course of the patient from initial presentation to long-term remission, divided into two distinct phases. (Upper panel) illustrates the diagnostic delay: the patient initially presented with severe iron-deficiency anemia (Hb 6.9 g/dL) and non-specific endoscopic findings, requiring a second endoscopy with deep biopsy months later to confirm collagenous gastritis when anemia recurred (Hb 10.2 g/dL). (Lower panel) details the treatment phase starting at week 0 with prednisone, omeprazole, and iron. Note the dissociation between clinical recovery and histological abnormalities at week 16, despite hemoglobin normalization (13.9 g/dL). Long-term management successfully achieved an asymptomatic state off steroids.

## Discussion

Collagenous gastritis (CG) is a rare inflammatory gastric disease and an uncommon but important etiology of IDA in children. We discuss a case of an 11-year-old boy with recurrent, transfusion-dependent IDA who's initial EGD showed erosive gastritis, but who was diagnosed with CG after repeat EGD and histologic evaluation. His anemia recurred despite PPI therapy and iron supplementation and was controlled only after oral corticosteroids were added. However, follow-up EGD showed no histologic improvement. This case highlights the diagnostic difficulty of pediatric CG and the therapeutic management for clinical improvement.

Clinical symptoms are variable with the pediatric CG presenting with abdominal pain, vomiting, fatigue, and IDA as in this case, and the adult type presenting with diarrhea due to concurrent involvement of the large intestine (5–8). Endoscopic findings may include gastric mucosal nodularity, erythema, erosions, hemorrhage, and ulcerations (1, 4, 9). Because these presentations are nonspecific, CG may be missed during the initial evaluation, especially when the presenting manifestation is anemia rather than gastrointestinal symptoms. In our case, the initial EGD revealed erosive nodular gastritis, and the diagnosis was determined only after recurrent IDA necessitated a repeat EGD. This clinical course promotes the suspicion for CG in children with recurrent IDA and nodular/erosive endoscopic changes.

The current characteristic histological finding of CG is subepithelial thickening of the collagen band, at least 10 mm in thickness (10). However, collagen deposition may be patchy in early disease, and routine hematoxylin-eosin staining may not suffice for diagnosis. In our patient's first biopsy, the diagnosis of childhood CG was not considered, and only routine endoscopic biopsy and staining were performed. Consequently, the pathology showed only chronic active inflammation. Assuring proper sampling from multiple gastric sites, biopsies of the surrounding nodular lesions, and special stains, such as Masson trichrome stain, will maximize diagnostic sensitivity and ensure an accurate diagnosis (4, 8, 11).

Currently, there are no accepted therapeutic standards for pediatric CG, as it is complex and individualized. Treatment often focuses on symptom control and iron therapy for anemia. For our patient, a combination therapy of iron supplementation and PPI was insufficient until the addition of prednisone ultimately led to resolution of symptoms and anemia, despite persistence of histopathologic abnormalities. In the case series reported by Beinvogl et al., pediatric patients received oral iron supplementation along with various medications, including PPI and steroids. They discovered that anemia responds well to iron supplementation in all patients, but there was no convincing evidence that any one intervention was beneficial (3). In another series reported by Emiliani et al., 4 patients were treated with a 6–8-week course of oral budesonide, but none showed endoscopic or histological improvement (1). A large study from Mayo Clinic reported by Choung et al. found that a mix of adult and pediatric patients with CG were treated with topically targeted budesonide (TTB), 89% of whom had a clinical response to TTB and 88% had a histologic response, indicating that TTB therapy produced clinical and histologic improvement (12). Although topical budesonide is associated with fewer systemic side effects, oral prednisone was selected in our case due to the severity of the patient's anemia and the need for rapid clinical remission, consistent with standard clinical practices for severe cases in our setting. Some literature suggests IDA most likely results from damage to dilated capillaries entrapped in the subepithelial collagen band. The observed symptomatic improvement despite persistent histological abnormalities suggests a clinico-histological dissociation. We think that corticosteroids effectively suppressed the active inflammation and stabilized the microvasculature, leading to symptom relief and anemia correction. However, the subepithelial collagen deposition, representing chronic structural remodeling, appears more refractory to treatment and persists even after clinical remission (4, 13). Hence, we highlighted the need to add corticosteroid therapy when IDA recurs despite iron supplementation and PPI, particularly if transfusion is required. Meanwhile, corticosteroids should still be used cautiously in pediatric patients to avoid systemic adverse effects or opportunistic infection.

The current literature emphasizes managing pediatric CG by treating symptoms to prevent complications and improve the patient's quality of life (6, 14). In our case, prednisone may stabilize hemoglobin levels and improve endoscopic appearance without inducing histologic remission. Case series analyses have shown that histological findings persist despite clinical improvement, underscoring the importance of close follow-up and biopsy to ensure histological resolution (1, 14). Based on our patient's clinical course, we recommend a stratified approach. For this patient, and generally for CG, the primary follow-up endpoints should be symptom control and hematologic stability rather than complete histological resolution, given the frequent clinico-histological dissociation. Given the chronicity of pediatric CG, this is vital for a deeper understanding of disease mechanisms and for standardizing disease management.

## Conclusions

Given the potential for recurrent anemia and blood transfusion from pediatric CG, pediatricians should be aware of the clinical signs of this rare disease to expedite implementation of proper therapy. This case also highlights the need to elucidate disease mechanisms to standardize disease management.

## Data Availability

The original contributions presented in the study are included in the article/Supplementary Material, further inquiries can be directed to the corresponding author.

## References

[1] Emiliani M Heintz D Izaguirre M. Collagenous gastritis: a cause of anemia and gastric nodularity in the pediatric population. JPGN Rep. (2026) 7(2):231–35. 10.1002/jpr3.70156 42110143 PMC13151020

[2] Broekaert IJ Assa A Borrelli O Saccomani MD Homan Mž Martin-de-Carpi J. Approach to anaemia in gastrointestinal disease: a position paper by the ESPGHAN gastroenterology committee. J Pediatr Gastroenterol Nutr. (2025) 80(3):510–32. 10.1002/jpn3.12454 39783775 PMC11874238

[3] Beinvogl BC Goldsmith JD Verhave M. Pediatric collagenous gastritis: clinical and histologic outcomes in a large pediatric cohort. J Pediatr Gastroenterol Nutr. (2021) 73(4):513–19. 10.1097/MPG.0000000000003212 34173792

[4] Assa A Borrelli O Broekaert I Saccomani MD Dolinsek J Martin-de-Carpi J. Helicobacter pylori-negative chronic gastritis in children: a systematic review. J Pediatr Gastroenterol Nutr. (2022) 74(5):956–67. 10.1097/MPG.0000000000003414 35175996

[5] Ma C Park JY Montgomery EA Arnold CA McDonald OG Liu T-C. A comparative clinicopathologic study of collagenous gastritis in children and adults: the same disorder with associated immune-mediated diseases. Am J Surg Pathol. (2015) 39(6):802–12. 10.1097/PAS.0000000000000441 25871617

[6] Blackmore C Leach M. Collagenous gastritis: a cause of pediatric iron deficiency anemia. ACG Case Rep J. (2023) 10(2):e01000. 10.14309/crj.0000000000001000 36891181 PMC9988283

[7] Romano M Plott N Galligan A Khalaf R. Literature review and a relevant case of pediatric collagenous gastritis: a rare but important etiology of iron-deficiency anemia. JPGN Rep. (2023) 4(4):e351. 10.1097/PG9.0000000000000351 38034434 PMC10684157

[8] Agrawal P Bhattar K Rojas C Larson J. Pediatric collagenous gastroduodenitis: a rare cause of iron-deficiency anemia. Cureus. (2024) 16(11):e72939. 10.7759/cureus.72939 39498423 PMC11532023

[9] Davis BG Goldman ML Cherny SC Lam AY Fox JM. Collagenous gastritis: a rare cause of hemorrhagic shock. ACG Case Rep J. (2025) 12(7):e01762. 10.14309/crj.0000000000001762 40626264 PMC12233915

[10] Li J Tanager K Setia N. Collagenous gastritis: clinical features, histologic correlates and unanswered questions. Histopathology. (2025) 87(6):789–801. 10.1111/his.15542 40911031 PMC12605687

[11] Lee YJ Lee M Kim DJ Lee S Hong J. Three case reports of collagenous gastritis in children: lessons for an endoscopic and histologic approach to mucosal nodularity of the stomach. Medicine (Baltimore). (2019) 98(11):e14870. 10.1097/MD.0000000000014870 30882690 PMC6426568

[12] Choung RS Sharma A Chedid VG Absah I Chen ZE Murray JA. Collagenous gastritis: characteristics and response to topical budesonide. Clin Gastroenterol Hepatol. (2022) 20(9):1977–85.e1. 10.1016/j.cgh.2021.11.033 34864160

[13] Matta J Alex G Cameron DJS Chow CW Hardikar W Heine RG. Pediatric collagenous gastritis and colitis: a case series and review of the literature. J Pediatr Gastroenterol Nutr. (2018) 67(3):328–34. 10.1097/MPG.0000000000001975 29601434

[14] Wilson KT Rizvi AH Turner KO. Rare concurrent findings with collagenous gastroduodenitis in a pediatric patient. ACG Case Rep J. (2026) 13(7):e02230. 10.14309/crj.0000000000002230 42383211 PMC13318076

